# The effect of heat stress on frame switch splicing of X-box binding protein 1 gene in horse

**DOI:** 10.5713/ajas.18.0757

**Published:** 2019-01-04

**Authors:** Hyo Gun Lee, Saichit Khummuang, Hyun-Hee Youn, Jeong-Woong Park, Jae-Young Choi, Teak-Soon Shin, Seong-Keun Cho, Byeong-Woo Kim, Jakyeom Seo, Myunghoo Kim, Tae Sub Park, Byung-Wook Cho

**Affiliations:** 1 Department of Animal Science, College of Natural Resources and Life Sciences, Pusan National University, Miryang 50463, Korea; 2 Graduate School of International Agricultural Technology and Institute of Green-Bio Science and Technology, Seoul National University, Pyeongchang 25354, Korea

**Keywords:** Thoroughbred, Heat Stress, X-box Binding Protein 1, Quantitative Real-time Polymerase Chain Reaction (qRT-PCR)

## Abstract

**Objective:**

Among stress responses, the unfolded protein response (UPR) is a well-known mechanism related to endoplasmic reticulum (ER) stress. ER stress is induced by a variety of external and environmental factors such as starvation, ischemia, hypoxia, oxidative stress, and heat stress. Inositol requiring enzyme 1α (IRE1α)-X-box protein 1 (XBP1) is the most conserved pathway involved in the UPR and is the main component that mediates IRE1α signalling to downstream ER-associated degradation (ERAD)- or UPR-related genes. XBP1 is a transcription factor synthesised via a novel mechanism called ‘frame switch splicing’, and this process has not yet been studied in the horse *XBP1* gene. Therefore, the aim of this study was to confirm the frame switch splicing of horse *XBP1* and characterise its dynamics using Thoroughbred muscle cells exposed to heat stress.

**Methods:**

Primary horse muscle cells were used to investigate heat stress-induced frame switch splicing of horse *XBP1*. Frame switch splicing was confirmed by sequencing analysis. *XBP1* amino acid sequences and promoter sequences of various species were aligned to confirm the sequence homology and to find conserved cis-acting elements, respectively. The expression of the potential XBP1 downstream genes were analysed by quantitative real-time polymerase chain reaction.

**Results:**

We confirmed that splicing of horse *XBP1* mRNA was affected by the duration of thermal stress. Twenty-six nucleotides in the mRNA of *XBP1* were deleted after heat stress. The protein sequence and the *cis*-regulatory elements on the promoter of horse *XBP1* are highly conserved among the mammals. Induction of putative downstream genes of horse XBP1 was dependent on the duration of heat stress. We confirmed that both the mechanisms of *XBP1* frame switch splicing and various binding elements found in downstream gene promoters are highly evolutionarily conserved.

**Conclusion:**

The frame switch splicing of horse *XBP1* and its dynamics were highly conserved among species. These results facilitate studies of ER-stress in horse.

## INTRODUCTION

Organisms have evolved impressive mechanisms to mitigate the negative effects of external stressors, including the unfolded protein response (UPR). The UPR is a well- known mechanism induced by endoplasmic reticulum (ER)-stress. When an organism is exposed to ER stress, UPR-related pathways are activated by three sensor proteins which exist in the ER: activating transcription factor-6 (ATF6), protein kinase R (PKR)-like endoplasmic reticulum kinase (PERK), and inositol requiring enzyme 1α (IRE1α) [1]. Binding immunoglobulin protein (BiP), also known as 78 kDa glucose-regulated protein (GRP78), is a lumen-resident molecular chaperone which is markedly up-regulated under ER-stress, dissociating from the three sensor proteins to activate each UPR pathway when unfolded and misfolded proteins are accumulated in the ER.

Among them, IRE1α-X-box protein 1 (XBP1) is the most conserved machinery utilised in the UPR. IRE1α is a transmembrane protein which self-activates via homo-dimerization and transphosphorylation during the UPR. Activated IRE1α functions similarly to endoribonuclease facilitating the splicing of *XBP1* mRNA in the cytosol [2,3]. This form of splicing is unconventional, as most pre-mRNAs are frequently the subject of spliceosome-dependent splicing in the nucleus [4]. Mammalian *XBP1* mRNA contains two characteristic stem-loop structures in which IRE1α-dependent splicing occurs, removing 26 nucleotides and exposing the transcriptional activation domain. As a consequence, translation of the spliced *XBP1* (*sXBP1*) produces a longer protein which functions as a transcription factor [3]. This novel splicing mechanism is called ‘frame switch splicing’ to distinguish it from conventional splicing occurring within the nucleus [5]. In contrast to the un-spliced form of XBP1 (usXBP1), sXBP1 is translocated into the nucleus following synthesis, where it induces the upregulation of genes related to ER-associated degradation (ERAD). ER homeostasis is thus maintained by the refolding and degradation of unfolded or misfolded proteins by processes facilitated by the IRE1α-XBP1 pathway.

The DNA-binding properties of sXBP1 are also important in the mediation of proper UPR function under the conditions of ER-stress. There are four evolutionarily conserved binding sites which can be found in the promoter region of UPR related genes, known as: ER stress response element (ERSE, consensus sequence: CCAAT-N9-CCACG), UPR element (UPRE, consensus sequence: TGACGTGG/A), ERSE-II (consensus sequence: ATTGG-N1-CCACGT), and UPRE-II (consensus sequence ATTGGTCCGCGT). Among these elements, ERSE has been well studied so as to ascertain the exact function of XBP1. The consensus sequence of ERSE is CCAAT-N9-CCACG [6,7]. Both ATF6 and XBP1, two basic leucine zipper proteins related to the UPR, can bind to the CCACG region of ERSE following the binding of NF-Y to the CCAAT region [3,8]. In response to ER stress, ER chaperone genes are transcriptionally regulated by the ATF6 and IRE1α-XBP1 pathways. ERSE is also found in the promoter region of *XBP1*, suggesting that positive feedback of *XBP1* transcription can sustain the function of XBP1 during the UPR.

Thoroughbred is one of the most adaptive horse breeds when considering racing performance, and is commonly used as a representative exercise model in the field of exercise physiology. The muscles of horses are subjected to higher thermal stress after exercise than other tissues [9], and although it is known that ER-stress is induced by heat stress, the horse *XBP1* gene has not yet been studied. Thus, the purpose of this study is to investigate whether the horse *XBP1* gene is evolutionarily conserved and to characterise its dynamics using Thoroughbred muscle cells exposed to heat stress.

## MATERIALS AND METHODS

### Tissue sampling

A skeletal muscle tissue biopsy was performed on the leg of a neonatal Thoroughbred to allow the cultivation of horse muscle cells. All materials and methods were approved by the Pusan National University-Institutional Animal Care and Use Committee (Approval Number: PNU-2015-0864).

### Primary horse muscle cell culture and heat stress

Horse muscle cells were maintained and sub-passaged in DMEM (Gibco, Grand Island, NY, USA) and were supplemented with 10% foetal bovine serum (Invitrogen, Carlsbad, CA, USA) and 1% antibiotic-antimycotic (ABAM; Invitrogen, USA). The cells were cultured in a humidified atmosphere with 5% CO_2_ at 37°C. Routine fluid renewals were performed 3 times per week. At 70% to 80% confluence, cells were gently washed twice with phosphate-buffered saline and harvested using 0.05% trypsin-ethylenediaminetetraacetic acid (Welgene, Gyeongsan, Korea) for total RNA extraction. To apply heat shock, the 70% to 80% confluent horse muscle cells were transferred to an incubator at a temperature of 40°C for 1 h and 4 h followed by a 4 h recovery period in an atmosphere of 5% CO_2_.

### RNA extraction and cDNA synthesis

Total RNA was extracted from horse muscle cells using TRIzol (Invitrogen, Karlsruhe, Germany) according to the manufacturer’s instructions. The purity of the extracted RNA was confirmed by measuring absorbance at 230 nm and 260 nm using a spectrophotometer (ND-1000, Nanodrop Technologies Inc., Wilmington, DE, USA), and RNA with a purity (OD value of 230 nm/260 nm) greater than 1.8 was selected for further analysis and stored at −70°C until the experiment was carried out.

To synthesise cDNA, 1 μg of RNA and 1 μL each of oligo-dT (Invitrogen, Waltham, MA, USA) and RNase-Free Water were added. The RNA was denatured at 80°C for 3 min and cDNA was synthesised using 4 μL of 5×RT (reverse transcription) buffer, 5 μL of 2 mmol/L dNTPs, 0.5 μL of RNase inhibitor, and 1 μL of M-MLV (Moloney-murine leukaemia virus) RT (Promega, Madison, WI, USA).

### Reverse transcription polymerase chain reaction and real time-quantitative polymerase chain reaction

The NCBI (http://www.ncbi.nlm.nih.gov) and the Ensembl Genome Browser (www.ensembl.org) were utilised to retrieve gene sequence information. The primers used in the amplification of the genes (Table 1) were synthesised using the PRIMER3 software (http://bioinfo.ut.ee/primer3-0.4.0/). Reverse transcription polymerase chain reaction (RT-PCR) and real-time quantitative PCR (qPCR) were carried out using a C1000 Thermal Cycler (Bio Rad, Hercules, CA, USA) using 25 μL of reaction solution to measure the relevant expression of target genes. The solution was created as follows: 2 μL of diluted cDNA (50 ng/μL) was added to 14 μL of SYBR green master mix (Bio Rad, USA) and 1 μL each of 5 pmol/μL diluted forward and reverse primers. The conditions used for the real-time qPCR were as follows: initial denaturation at 94°C for 10 mins followed by 40 cycles of denaturation at 94°C for 10 s, annealing at 60°C for 10 s, and extension at 72°C for 30 s. All measurements were carried out in triplicate for each specimen, and the 2^−ΔΔCt^ method [10] was used to determine relative gene expression. The relative expression of target genes was normalised with glyceraldehyde 3-phosphate dehydrogenase.

### Sequencing

Agarose gel electrophoresis was performed using the PCR products amplified by the XBP1 primer set (Forward: 5′-AGC TCGAATGAGTGAGCTGG-3′, reverse: 5′-ATCCATGGGG AGAGGTTCTGG-3′) (Table 1). The clear PCR bands were then removed from the gel using a LaboPass gel extraction kit (COSMO GENETECH, Seoul, Korea). The amplicons were ligated using the pGEM-T easy vector (Promega, USA) for DNA sequencing with T7 (5′-TAATACGACTCACTATA-3′) and SP6 (5′-CTATAGTGTCACCTAAAT-3′) primers. Sequencing results were analysed against annotated horse genomes using the basic local alignment search tool (BLAST).

### Secondary structure analysis

Secondary structure prediction of the deleted nucleotides in *XBP1* mRNA was performed using Geneious version (6.0.6) (http://www.geneious.com) [11]. The predicted RNA folding structures were created using Turner’s energy model at 37°C.

### Alignment of protein and promoter sequences

The XBP1 protein sequences of the different species including homo sapiens (ENSG00000100219), equus caballus (ENSECAG 00000014780), mus musculus (ENSMUSG00000020484), gallus gallus (ENSGALG00000005796), xenopus laevis (ENSX ETG00000027416), and danio rerio (ENSDARG00000035622) were retrieved from Ensembl release 92 (http://asia.ensembl.org) and aligned using Geneious version (6.0.6) (http://www.geneious.com) [11]. Approximately 200 upstream nucleotides from the transcription start site of *XBP1* in the five species (homo sapiens, macaca mulatta, equus caballus, bos taurus, and mus musculus) were retrieved from the NCBI database. Putative transcription factors, which bind to the promoter of *XBP1*, were annotated using EMBOSS: tfscan based on the TRANSFAC database. The upstream sequences of horse *BiP*, *GRP94*, and homocysteine-responsive endoplasmic reticulum-resident ubiquitin-like domain member 1 protein (*Herpud1*) were retrieved to search the binding sites of XBP1.

### Statistical analysis

Means and standard deviations were calculated using Microsoft Excel. The statistical significance of the results (* p<0.05, ** p<0.01, or *** p<0.001) was assessed using one-way analysis of variance followed by post-hoc comparison (Tukey’s honestly significant difference test) using the Prism 5 program (San Diego, CA, USA).

## RESULTS

### Heat mediated splicing of *XBP1* in horse muscle cells

**Figure 1:**
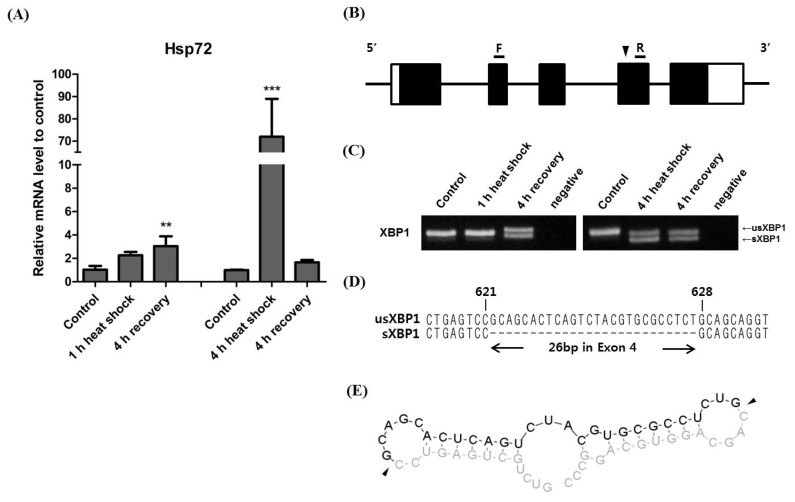
Heat stress mediated *XBP1* splicing in Thoroughbred muscle derived cells. (A) Time course induction of *Hsp72*. Horse muscle cells were incubated for 1 h and 4 h at 40°C followed by 4 h recovery shock at 37°C. *Hsp72* expression was analysed by real-time qPCR using cDNA after both the 1 h heat shock (one-way ANOVA, p = 0.011) and 4 h heat shock (one-way ANOVA, p = 0.0002). Asterisk denotes statistical signi_cance (** p<0.01, *** p<0.001) of post-hoc comparisons (Tukey’s HSD) with the control for each heat shock duration. The results were normalised to *GAPDH*. Error bars represent the standard deviation (n = 3). (B) Schematic presentation of the horse *XBP1* gene structure. White and black boxes indicate untranslated regions and exons, respectively. The location of the forward (F) and reverse (R) primers for detection of the spliced *XBP1* mRNA are shown. The arrow head indicates the location of the spliced region relative to the primers. (C) *XBP1* mRNA splicing after both the 1 h and 4 h heat shock followed by a 4 h recovery period. RT-PCR was used to detect *usXBP1* and *sXBP1* mRNA. Upper bands (294 bp) and lower bands (268 bp) indicate *usXBP1* and *sXBP1*, respectively. (D) Nucleotide sequences of cDNA corresponding to *usXBP1* and *sXBP1* transcripts around the splicing sites. 26 bp deletion occurred in exon 4 of *XBP1* mRNA after heat shock. (E) The predicted secondary folding structure of the deleted region in the *XBP1* mRNA at 37°C depicted by Turner’s energy model. The cleavage sites in *XBP1* mRNA are indicated by arrow heads. XBP1, X-box binding protein 1; Hsp72, heat shock 70 kDa protein 1; qPCR, quantitative real-time polymerase chain reaction; ANOVA, analysis of variance; HSD, honestly signi_cant difference; *GAPDH*, glyceraldehyde 3-phosphate dehydrogenase; RT-PCR, Reverse transcription polymerase chain reaction; usXBP1, un-spliced XBP1; sXBP1, spliced XBP1.

To induce *XBP1* splicing, horse muscle cells were subjected to a 1 h and 4 h heat shock at 40°C followed by 4 h of recovery at 37°C [12]. *Hsp72*, a member of the heat shock protein 70 family and a chaperone protein used as a marker of heat stress, immediately increased during the 1 h heat shock, and increased significantly during the 4 h heat shock. *Hsp72* in cells subjected to the 4 h heat shock subsequently decreased during the 4 h recovery period (Figure 1A). To detect the *XBP1* splicing site, we predicted the deleted region based on the human *XBP1* splicing site and designed a primer set to produce a 294 bp PCR amplicon encompassing exon 3 of horse *XBP1* (Figure 1B). As a result, we successfully detected a clear band in the 294 bp PCR product indicative of *XBP1* splicing. Interestingly, in cells exposed to the 1 h heat shock, *XBP1* splicing was not induced during heat shock exposure but was induced during the 4 h recovery period. In cells exposed to the 4 h heat shock, *XBP1* splicing was clearly induced (Figure 1C). These results show that heat mediated *XBP1* splicing in horse muscle cells is only induced by more than 1 h of heat shock and that the spliced form of *XBP1* mRNA persists until after the 4 h recovery period. Next, we cloned the bands of both un-spliced (*usXBP1*) and spliced XBP1 (*sXBP1*) and conducted sequencing analysis to identify the splicing regions, the results of which showed that a region consisting of 26 nucleotides within exon 4 of horse *XBP1* were deleted (Figure 1D). Furthermore, we predicted the secondary structure of *sXBP1* using the mRNA sequence from around the deleted region of *XBP1*. The result shows that there are two characteristic loop structures defined within the conserved IRE1α cleavage sites [5] (Figure 1E). Taken together, the results of these analyses confirm that the horse *XBP1* gene is subjected to frame shift splicing.

### Heat stress-induced changes in the ratio of *sXBP1* to *usXBP1* in horse muscle cells

**Figure 2:**
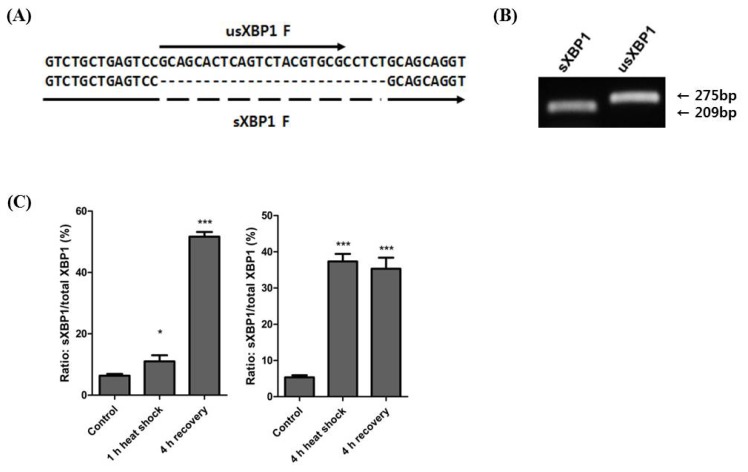
Expression of *usXBP1* and *sXBP1* mRNA after heat shock exposure. (A) Design of forward primers detecting the speci_c forms of *XBP1* mRNA. Black arrows indicate each forward primer. Dotted line shows the deleted region of *sXBP1*. (B) RT-PCR analysis for the con_rmation of the primer sets detecting the speci_c forms of *XBP1* mRNA. (C) The ratio of *sXBP1* to total *XBP1* mRNA was evaluated by real-time qPCR of cDNA obtained after the 1 h heat shock (n = 3, one-way ANOVA, p<0.0001) and 4 h heat shock (n = 3, one-way ANOVA, p<0.0001). Asterisk denotes statistical signi_cance (** p<0.01, *** p<0.001) of post-hoc comparisons (Tukey’s HSD) with the control for each heat shock duration. The results were normalised to *GAPDH*. Error bars represent the standard deviation (n = 3). XBP1, X-box binding protein 1; usXBP1, un-spliced XBP1; sXBP1, spliced XBP1; RT-PCR, Reverse transcription polymerase chain reaction; qPCR, quantitative real-time polymerase chain reaction; ANOVA, analysis of variance; HSD, honestly signi_cant difference; *GAPDH*, glyceraldehyde 3-phosphate dehydrogenase.

To understand *XBP1* splicing during exposure to heat stress, we designed primer sets to specifically detect *usXBP1* and *sXBP1* transcripts (Figure 2A). We confirmed the specificity of primer amplification by gel electrophoresis using the cDNA from horse muscle cells exposed to the 4 h heat shock (Figure 2B). Following the 1 h heat shock, the ratio of *sXBP1* transcripts to total *XBP1* transcripts slightly increased, and the ratio was significantly increased after the 4 h recovery period. Conversely, the ratio had significantly increased after the 4 h heat shock and remained high until the 4 h recovery period (Figure 2C). These results indicate that prolonged heat stress affects *XBP1* splicing.

### Sequence homology of horse XBP1

**Figure 3:**
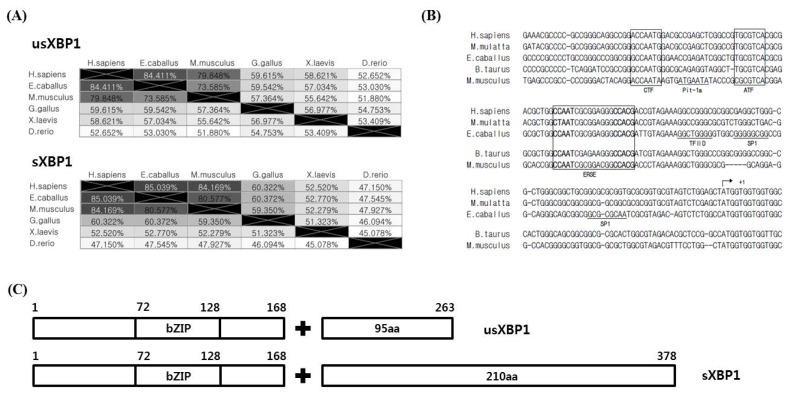
Sequence conservation of XBP1. (A) Protein sequence homology of *sXBP1* and *usXBP1*. The values in the matrix indicate the similarity of protein sequences between the two species. (B) Alignment of promoter sequences between species. An arrow indicates the transcription start site (+1) of *XBP1*. Boxes denote the conserved binding sites of transcription factors in the *XBP1* promoter region. Bolded sequences indicate the binding sequences of nuclear factor (NF-Y) (CCAAT), activating transcription factor 6 (ATF6), and XBP1 (CCACG). Putative binding sites of transcription factors are underlined. (C) Schematic representation of the structure of usXBP1 and sXBP1 with amino acid number. The protein length of sXBP1 is longer than that of usXBP1. XBP1, X-box binding protein 1; sXBP1, spliced XBP1; usXBP1, un-spliced XBP1; Pit-1a, POU class 1 homeobox 1; ATF, activating transcription factor; bZIP, basic leucine zipper domain; aa, amino acid.

The XBP1 protein sequence homology was investigated using human, horse, mouse, chicken, frog, and zebrafish sequences. Human usXBP1 and sXBP1 showed the highest similarity to horse usXBP1 and sXBP1 (usXBP1: 84.411% and sXBP1: 85.039%). Birds, amphibians, and fish showed relatively lower similarities in their XBP1 sequences than those seen in mammals (Figure 3A). Next, the human, monkey, horse, cattle, and mouse promoter sequences were aligned to search for conserved transcription factor binding elements. We found that the CCAAT box-binding transcription factor, ATF, and ERSE were highly conserved among taxonomic groups. Additionally, the binding sites for transcription factor II D and specificity protein 1 in the horse *XBP1* promoter were predicted (Figure 3B).

According to a study of human *XBP1* genes, *sXBP1* encodes longer proteins than *usXBP1*, and the frame-shifted part of sXBP1 exhibits higher transcriptional activator activity [3]. When usXBP1 and sXBP1 were translated to identify the open reading frame, we noted that splicing of *XBP1* replaced the C-terminal portion of XBP1 with 210 amino acids. Using protein domain prediction tools on horse XBP1 protein sequences showed that the unchanged part of XBP1 contains a basic leucine zipper domain. We could predict any domain based on the C-terminal part of horse sXBP1 (Figure 3C), however, based on the protein sequence homology of horse sXBP1, it is reasonable to assume that the replaced part of the C-terminal has a transcription activator domain. Taken together, these results indicate that not only the cis-regulatory elements in the promoter and protein sequence of XBP1, but also the protein domains of XBP1 are highly conserved among the studied groups.

### Expression of downstream genes of XBP1

**Figure 4:**
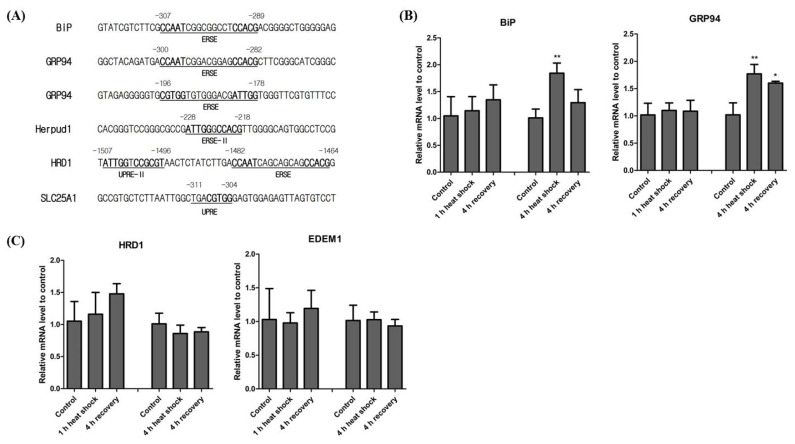
Expression of the downstream genes of XBP1 after heat shock exposure. (A) Conserved binding sequences of the ERSE and ERSE-II on *BiP*, *GRP94*, and *Herpud1* promoter. The numbers above the sequences denote the position of the transcription start site of each gene. Bolded sequences indicate the binding sequences of nuclear factor (NF-Y) (CCAAT and ATTGG), activating transcription factor 6 (ATF6), and XBP1 (CCACG and CGTGG). (B) Expression of *BiP* and *GRP94* after heat shock. *BiP* (one-way ANOVA, 1 h heat shock: p = 0.5033, 4 h heat shock: p = 0.0062) and *GRP94* (one-way ANOVA, 1 h heat shock: p = 0.8463, 4 h heat shock: p = 0.0031) expression was investigated using real-time qPCR. (C) Expression of *HRD1* (one-way ANOVA, 1 h heat shock: p = 0.4513, 4 h heat shock: p = 0.3608) and *EDEM1* (one-way ANOVA, 1 h heat shock: p = 0.7198, 4 h heat shock: p = 0.7406) were investigated by real-time qPCR. The results were normalised to *GAPDH*. Error bars represent the standard deviation (n = 3). XBP1, X-box binding protein 1; ERSE, endoplasmic reticulum-stress response element; BiP, binding immunoglobulin protein; GRP94, heat shock protein 90 kDa beta member 1; Herpud1, homocysteine-responsive endoplasmic reticulum-resident ubiquitin-like domain member 1 protein; UPRE, UPR element; ANOVA, analysis of variance; qPCR, quantitative real-time polymerase chain reaction; HRD1, E3 ubiquitin-protein ligase synoviolin; EDEM1, endoplasmic reticulum degradation enhancing alpha-mannosidase-like protein 1; SLC25A1, solute carrier family 25 member 1.

XBP1 contains several distinct binding elements relating to the promoter regions of several downstream genes, including ERSE, ERSE II, UPRE, and UPRE-II. *BiP* and *GRP94* are two representative genes regulated by XBP1 [6,7]. Additionally, *Herpud1*, a molecular chaperone for proteins in the ER, contains both ERSE and ERSE-II in its promoter region [13]. Thus, Herpud1 is likely induced by various ER-stress reagents [14]. We thus investigated both ERSE and ERSE-II present in horse *BiP*, *GRP94*, and *Herpud1* promoters. Horse *BiP* and *GRP94* contain ERSE in a similar position to XBP1. Horse *GRP94* has an additional version of ERSE but in the reverse direction. In the horse *Herpud1* promoter, we also found evidence of ERSE-II (Figure 4A). Unlike humans, horse *Herpud1* does not contain ERSE. To understand heat mediated transcription of genes downstream of XBP1, we conducted real-time qPCR of genes containing ERSE, *BiP*, and *GRP94* in their promoter regions. We found that both *BiP* and *GRP94* transcription were not significantly changed after the 1 h heat shock, but after the 4 h heat shock *BiP* and *GRP94* transcription was significantly increased (Figure 4B). These results indicate that the expression of both *BiP* and *GRP94* is affected by the duration of heat stress.

It was initially assumed that UPRE was located in the promoter of ERAD genes such as E3 ubiquitin-protein ligase synoviolin (*HRD1*) and ER degradation enhancing alpha-mannosidase-like protein 1 (*EDEM1*) [15,16]. However, it was later confirmed that the human *HRD1* promoter contains functional versions of the UPRE, UPRE-II, and ERSE [17]. However, the binding site of XBP1 in the *EDEM1* promoter has not yet been studied. We could not find any evidence of UPRE being present in the horse *HRD1* and *EDEM1* promoter regions. Additionally, even though *HRD1* and *EDEM1* are solely regulated by the IRE1α-XBP1 pathway [16,18], UPRE-II and ERSE were only conserved in horse *HRD1*. Similar to studies of human *EDEM1*, we could not find any ER-stress related elements in the horse *EDEM1* promoter (Figure 4A). As ER-stress induced protein degradation is associated with the IRE1α-XBP1 pathway, we investigated the transcriptional induction of the ERAD-related genes, *HRD1* and *EDEM1*. The expression of both *HRD1* and *EDEM1* after both 1 h and 4 h of heat shock was not altered significantly (Figure 4C). These results suggest that neither 1 h nor 4 h of heat shock are sufficient to induce the ERAD system.

According to a recent study, the human citrate carrier (*CiC*) gene contains UPRE [19]. We searched the horse CiC homologue using the BLAST search tool. Horse clathrin heavy chain 2 isoform 6 (XP_023502409.1), also called solute carrier family 25 member 1 (SLC25A1), showed the highest protein sequence similarity to human CiC (query cover, 99%; identity, 97%). We also found that horse *SLC25A1* contains UPRE in the promoter region (Figure 4A).

These results clearly indicate that conserved XBP1 binding elements also exist in the horse promoter regions of genes downstream of XBP1. Additionally, as the transcriptional regulation of *BiP*, *GRP94*, *HRD1*, and *EDEM1* depends on the duration of heat stress, we can confirm that the refolding and degradation of unfolded and misfolded proteins is stress- and/or time-dependent.

## DISCUSSION

ER-stress induces a signalling network known as the UPR which mitigates the negative effects of ER-stress and works to maintain homeostasis. Recent research indicates that the UPR plays important roles in the physiological responses to various diseases, including neurological diseases [20], diabetes [21], cancer [22], and rheumatoid arthritis [23]. ER-stress is induced by a variety of external and environmental factors such as starvation, ischemia, hypoxia, oxidative stress, and heat stress.

Under the heat stress, Hsp72 functions as a molecular chaperone [24]. Exposure to 4 h of heat shock dramatically increased *hsp72* mRNA expression levels, and we can thus assume that the duration of heat stress affects the quantity of misfolded and unfolded proteins (Figure 1A). The mRNA expression level of *hsp72* was reduced after 4 h of recovery at 37°C. This is due to the presence of hsp72 protein at high concentrations resulting from the 4 h of heat shock. Conversely, spliced *XBP1* mRNAs were maintained at high concentrations even after 4 h of recovery in the 4 h heat shock treatment (Figure 1C, 2C). The prolonged persistence of spliced *XBP1* may be facilitated by IRE1α-XBP1 signalling and the XBP1 self-regulatory system [8,25] (Figure 3B).

When the IRE1α-XBP1 pathway is induced in response to ER-stress, sXBP1 regulates downstream genes such as *BiP*, *GRP94*, *Herpud1*, and *HRD1* [7,13,17]. ERSE, ERSE-II, UPRE, and UPRE-II have been identified to date [15,17], and our result shows that horse ER stress-related genes contain conserved ERSE, ERSE-II, UPRE, and UPRE-II sequences (Figure 4A). It was postulated that ERAD genes contain UPRE in their promoter regions. A partially palindromic sequence (TGAC GTGG/A) in ATF6α was initially found by binding site selection experiments [26]. However, it has been suggested that the consensus sequence of the ATF6α binding site in its UPRE is not for ATF6α but for XBP1 [3]. It has also been suggested that ATF6α produced at physiologically accurate concentrations is insufficient to induce the transactivation of the UPRE reporter due to its low affinity for the UPRE [15]. Interestingly, although UPRE has been intensively studied in yeast [27,28], UPRE was not found in mammalian promoter regions for a considerable time [15]. Recently, however, UPRE was found in the promoter region of the human *CiC* gene [19]. In this study, we located the UPRE sequence in horse *SLC25A* but not in ERAD genes such as *HRD1* and *EDEM1*. Therefore, it is likely that other ER-stress-related elements may compensate for the absence of UPRE in horse ERAD genes or perhaps a novel and unique mechanism exists. The differences between species in their functional ER-stress-related elements, including UPRE, should be studied in greater detail in the future.

As *XBP1* mRNA is spliced after ATF6α cleavage, the UPR undergoes a time-dependent transition from the ‘refolding only’ phase to the ‘refolding plus degradation’ phase [16]. Although XBP1 downstream genes contain consensus binding elements, their induction times differ according to their function. For example, EDEM shows increased delayed transcriptional induction when compared to BiP. Mouse embryonic fibroblasts devoid of IRE1α demonstrate that *EDEM* mRNA is not produced under ER-stress. Considering that physiologically accurate concentrations of ATF6α could not induce UPRE, UPRE is likely solely regulated by the IRE1α-XBP1 pathway [16]. Although horse *HRD1* and *EDEM1* do not contain UPRE, their expression was not altered to the same degree as *BiP* and *GRP94*, suggesting that 4 h of heat shock is not a sufficient duration to induce the ERAD system (Figure 4C). Therefore, our findings support a time-dependent transition model. In the future, the progression of the IRE1α-XBP1 pathway and the level of gene expression relative to the duration and intensity of heat stress should be investigated.

In conclusion, we firstly investigated ER-stress in horse muscle cells subjected to mild heat stress and verified the existence of frame shift splicing in horse *XBP1*. We also confirmed that the genes related to the UPR and other associated regulatory elements were highly conserved among different animal groups. Our results provide valuable information for the continuation of ER stress-related studies in horse in relation to various environmental stresses.

## Figures and Tables

**Table 1:** Primer sets used in this study

Primer name	Primer sequence (5′ to 3′)	Tm (°C)	Product size (bp)
Hsp72 F	CGACCTCAACAAGAGCATCA	60	213
Hsp72 R	AAGATCTGCGTCTGCTTGGT		
Bip F	TCTCTGAGACCCTGACTCGG	58	217
Bip R	ATCTGGGTTTATGCCACGGG		
EDEM1 F	TCTGTGGACAAGCACCTTCG	58	232
EDEM1 R	GGAGAGCCGGTCAGATCAAG		
GRP94 F	CTCCTGCGATCAGAAGGGAC	58	120
GRP94 R	TCGACTTCATCGTCAGCTCG		
HRD1 F	GTGATGGGCAAGGTGTTCTT	58	274
HRD1 R	GGATGCCCAAGAGGAACATA		
XBP1 F	AGCTCGAATGAGTGAGCTGG	58	294
XBP1 R	ATCCATGGGGAGAGGTTCTGG		
sXBP1 F	GTCTGCTGAGTCCGCAGCAGGT	58	209
sXBP1 R	TGGGTCCTTCTGGGTAGACC		
usXBP1 F	GCAGCACTCAGTCTACGTGCG	58	275
usXBP1 R	CAGCTTGGCTGATGACGTCCC		
GAPDH F	GGTGAAGGTCGGAGTAAACG	60	106
GAPDH R	AATGAAGGGGTCATTGATGG		

Hsp, heat shock protein; Bip, binding immunoglobulin protein; EDEM1, enhancing alpha-mannosidase-like protein 1; GRP, glucose-regulated protein; HRD1, E3 ubiquitin-protein ligase synoviolin; XBP1, X-box protein 1; sXBP1, spliced XBP1; usXBP1, un-spliced XBP1; GAPDH, glyceraldehyde 3-phosphate dehydrogenase.
